# Editorial: CAKUT in Children and Adolescents: Towards Better Understanding of Impact and Risk Reduction

**DOI:** 10.3389/fped.2022.863573

**Published:** 2022-04-18

**Authors:** Augustina Jankauskiene

**Affiliations:** Institute of Clinical Medicine, Faculty of Medicine, Vilnius University, Vilnius, Lithuania

**Keywords:** CAKUT, ultrasound, screening, biomarkers, genetics

Congenital anomalies of the kidney and urinary tract (CAKUT) are the most common congenital malformations and are also among the leading causes of end-stage kidney disease (ESKD) in the pediatric population. An absent or malformed kidney is a severe defect occurring early in gestation, whereas defects that occur later are generally less severe, such as obstruction, vesicoureteric reflux, posterior urethral valves, etc.

In this special CAKUT series, we have assembled contributions from 24 researchers from different parts of the world to provide the reader with a comprehensive and current update with four papers on the various aspects. The main challenges are the screening programs using ultrasound (US), which are used to detect severe abnormalities and take action, to settle upon a recommended time for screening or follow up, to discuss genetic testing possibilities and their clinical impact, and to look for combined malformations possibly detected via US or genetic testing results. Researchers are looking for new urine biomarkers to become additional tools for making important and timely surgical decisions and prevent invasive procedures for children.

The diversity of the malformations summarized by CAKUT is high, and there are numerous associated syndromes. The need to screen girls with the coincidence of a congenital solitary functioning kidney and Müllerian anomalies for uterine and vaginal anomalies has, however, only been described recently. Diagnosis of Müllerian malformations is therefore often delayed until after menarche when affected girls present with dysmenorrhea, intermenstrual bleeding, mucopurulent vaginal discharge, and/or abdominal pain as a result of obstruction. The authors van Dam et al. present four cases and stress the importance of abdominal US with a focus on Müllerian anomalies to be implemented in the routine clinical practice recommendations in the first weeks of life and/or in the pubertal age.

The importance of US is widely discussed, but the broad screening programs and the economic and medical burden are not yet fully evaluated. Liu et al. try to evaluate the value of US screening for CAKUT during the early postnatal period and compare this with antenatal US. Postnatal US scanning in 4,877 infants identified 7.4 % of CAKUT with urinary tract dilatation (UTD) being the most frequent, but a specific diagnosis was identified in 47 cases (0.96%) within the 6-month screening and follow-up program. It seems that UTD resolves with time, and this has spurred an open discussion on further follow-up tactics and the topic of whether all CAKUT has the same impact on further outcomes. Additionally, US evaluations may be prone to bias by different investigators.

Some CAKUT require surgical intervention; Kazlauskas et al. tried to establish the efficacy of US combined with urine biomarkers in differentiating patients with hydronephrosis who require surgical management from those who do not, avoiding invasive investigations.

Logistic regression model and stepwise model selection demonstrated that significant US parameters for the prediction of operation were mid-parenchymal thickness and anterior-posterior dilatation (APD) of the renal pelvis, while the calyceal dilatation, renal volume, and alternative grading systems used were not. Combined as the APD/mid-parenchymal ratio, the two parameters were used as a significant predicting factor for surgery and were independent of age.

Findings suggest that the APD/mid-parenchymal ratio can be a promising index for US. It demonstrated a substantial specificity of 89% and sensitivity of 67% in the prediction of surgery. The role of urine biomarkers was evaluated by combining US findings with urine albumin, β2-microglobulin (β2-M), and urinary neutrophil gelatinase-associated lipocalin (uNGAL); specificity and sensitivity increased to 94 and 71%, respectively, although insignificantly. The β2-M/creatinine ratio in voided urine was the most promising predictor in the study cohort. These findings advocate for further prospective studies on the development of the multivariable diagnostic protocol and support an idea to make hydronephrosis evaluation non-invasive, but they shed doubt on the use of urine biomarkers in everyday practice.

The genetic background of CAKUT remains unknown in the majority of cases. Paired box 2 (PAX2)-related disorder is an autosomal dominant genetic disorder associated with kidney and eye abnormalities, and it can result in ESKD. Chang et al. report three patients from two families with PAX2 mutations. Two patients were adults with chronic kidney disease without correct diagnoses, including one with ESKD and kidney transplantation. The third patient was a neonate in whom PAX2-related disorder manifested as oligohydramnios, coloboma, and renal failure that progressed to ESKD within 1 year after birth. This family example shows that phenotypes of PAX2 gene mutation were highly variable. Early detection with genetic counseling and guided clinical management might result in better outcomes for patients. With improved genetic sequencing techniques, more genetic abnormalities can be detected.

Still, many questions stay open. Further work on understanding the complex mechanisms involved in the pathogenesis of CAKUT is needed. Validated data from evidence-based multicenter studies might have an impact on quality of life for CAKUT patients with a more unified diagnostic and care management strategy starting already antenatally.

## Author Contributions

The author confirms being the sole contributor of this work and has approved it for publication.

## Conflict of Interest

The author declares that the research was conducted in the absence of any commercial or financial relationships that could be construed as a potential conflict of interest.

## Publisher's Note

All claims expressed in this article are solely those of the authors and do not necessarily represent those of their affiliated organizations, or those of the publisher, the editors and the reviewers. Any product that may be evaluated in this article, or claim that may be made by its manufacturer, is not guaranteed or endorsed by the publisher.

